# N6-methylandenosine-related lncRNAs play an important role in the prognosis and immune microenvironment of pancreatic ductal adenocarcinoma

**DOI:** 10.1038/s41598-021-97362-9

**Published:** 2021-09-08

**Authors:** YuHai Hu, YiPing Chen

**Affiliations:** Department of Hepatopancreatobiliary Surgery, Fujian Abdominal Surgery Research Institute, the First Affiliated Hospital of Fujian Medical University, Fujian, China

**Keywords:** Cancer genetics, Gastrointestinal cancer, Tumour biomarkers

## Abstract

Pancreatic ductal adenocarcinoma (PDAC) is a highly aggressive, fatal tumor. N6-methylandenosine (m6A) methylation is the major epigenetic modification of RNA including lncRNAs. The roles of m6A-related lncRNAs in PDAC have not been fully clarified. This study aims to assess gene signatures and prognostic value of m6A-related lncRNAs in PDAC. The Cancer Genome Atlas (TCGA) dataset and the International Cancer Genome Consortium (ICGC) dataset were explored to identify m6A-related lncRNAs. Univariate, least absolute shrinkage and selection operator (LASSO) and multivariate Cox regression were performed to construct the m6A-related lncRNAs prognostic riskscore (m6A-LPR) model to predict the overall survival (OS) in the TCGA training cohort. Kaplan–Meier curve with log-rank test and receiver operating characteristic (ROC) curve were used to evaluate the prognostic value of the m6A-LPR. Furthermore, the robustness of the m6A-LPR was further validated in the ICGC cohort. Tumor immunity was evaluated using ESTIMATE and CIBERSORT algorithms. A total of 262 m6A-related lncRNAs were identified in two datasets. In the TCGA training cohort, 28 prognostic m6A-related lncRNAs were identified and the m6A-LPR including four m6A-related lncRNAs was constructed. The m6A-LPR was able to identify high-risk patients with significantly poorer OS and accurately predict OS in both the TCGA training cohort and the ICGC validation cohort. Analysis of tumor immunity revealed that high-risk groups had remarkably lower stromal, immune, and ESTIMATE scores. Moreover, high-risk groups were associated with significantly higher levels of plasma B cells and resting NK cells infiltration, and lower levels of infiltrating resting memory CD4 T cells, monocytes, and resting mast cells. Our study proposed a robust m6A-related prognostic signature of lncRNAs for predicting OS in PDAC, which provides some clues for further studies focusing on the mechanism process underlying m6A modification of lncRNAs.

## Introduction

Pancreatic ductal adenocarcinoma (PDAC) is a major histological subtype of pancreatic cancer. The incidence of PDAC is rising and the five-year survival rate is less than 5% with no significant improvement in survival over the past 10 years^1,2^. Surgical resection offers the only potentially curative treatment, but 80% of patients with PDAC are not amenable to surgery at diagnosis^3^. The efficacy of systemic treatment is limited and the advent of targeted and immune therapies are promising strategies to address this challenge. Thus, it is urgent to investigate potential therapeutic targets for PDAC.

N6-methylandenosine (m6A) RNA methylation is the main epigenetic modification of messenger RNAs (mRNAs) and non-coding RNAs (ncRNAs)^4^, it has been confirmed to play critical regulatory roles in the modification of tumor RNAs^5^. m6A modifications are invertible and dynamical processes that are regulated by three kinds of m6A regulator, including methyltransferases (“writers”), signal transducers (“readers”) and demethylases (“erasers”)^6^, for example, METTL14, an m6A methyltransferase, catalyzes m6A RNA methylation with METTL3, and these two proteins form a stable METTL3–METTL14 complex that functions in cellular m6A deposition on mammalian nuclear RNAs^7^. Recent studies demonstrated that m6A modification involves the regulation of oncogenesis and tumor progression in PDAC^8–13^. ALKBH5 serves as a PDAC suppressor by regulating the posttranscriptional activation of PER1 through m6A abolishment^8^ and decreasing WIF-1 RNA methylation and mediating Wnt signaling^10^. Upregulation of METTL14 can promote the growth and metastasis of PDAC by decreasing PERP levels^12^.

Long non-coding RNAs (lncRNAs) regulate the biological functions of cells, including the proliferation, infiltration, and metastasis of certain tumor cells^14^, and dysregulation of lncRNAs had been reported to play a crucial role in the carcinogenicity of PDAC^9,15–17^. A recent study found that m6A reader IGF2BP2 regulates lncRNA DANCR to promote cancer stemness-like properties and pancreatic cancer pathogenesis^9^. Nevertheless, the full impact of m6A regulators on the aberrant lncRNAs expression in cancers is still unclear and few studies have been conducted to investigate the mechanisms underlying how lncRNAs are regulated by m6A modification to involve in the onset and development of PDAC. Therefore, understanding how m6A modifications of lncRNAs contribute to PDAC progression can help to identify novel biomarkers as potential therapeutic targets.

In this study, The Cancer Genome Atlas (TCGA) dataset (n = 140) and the International Cancer Genome Consortium (ICGC) dataset (n = 63) were explored to identify 262 m6A-related lncRNAs in patients with PDAC. Then we found that 28 prognostic m6A-related lncRNAs in the TCGA cohort and we constructed an m6A-related lncRNAs prognostic riskscore (m6A-LPR) model including four prognostic m6A-related lncRNAs to predict the overall survival (OS) of patients with PDAC. The relevance of the m6A-LPR with tumor immunity was also evaluated. Our results would be helpful to assess the prognosis of patients with PDAC and might offer the promise of individualized therapeutic interventions.

## Materials and methods

### Datasets, m6A-related genes and annotation of lncRNAs

**Figure 1:**
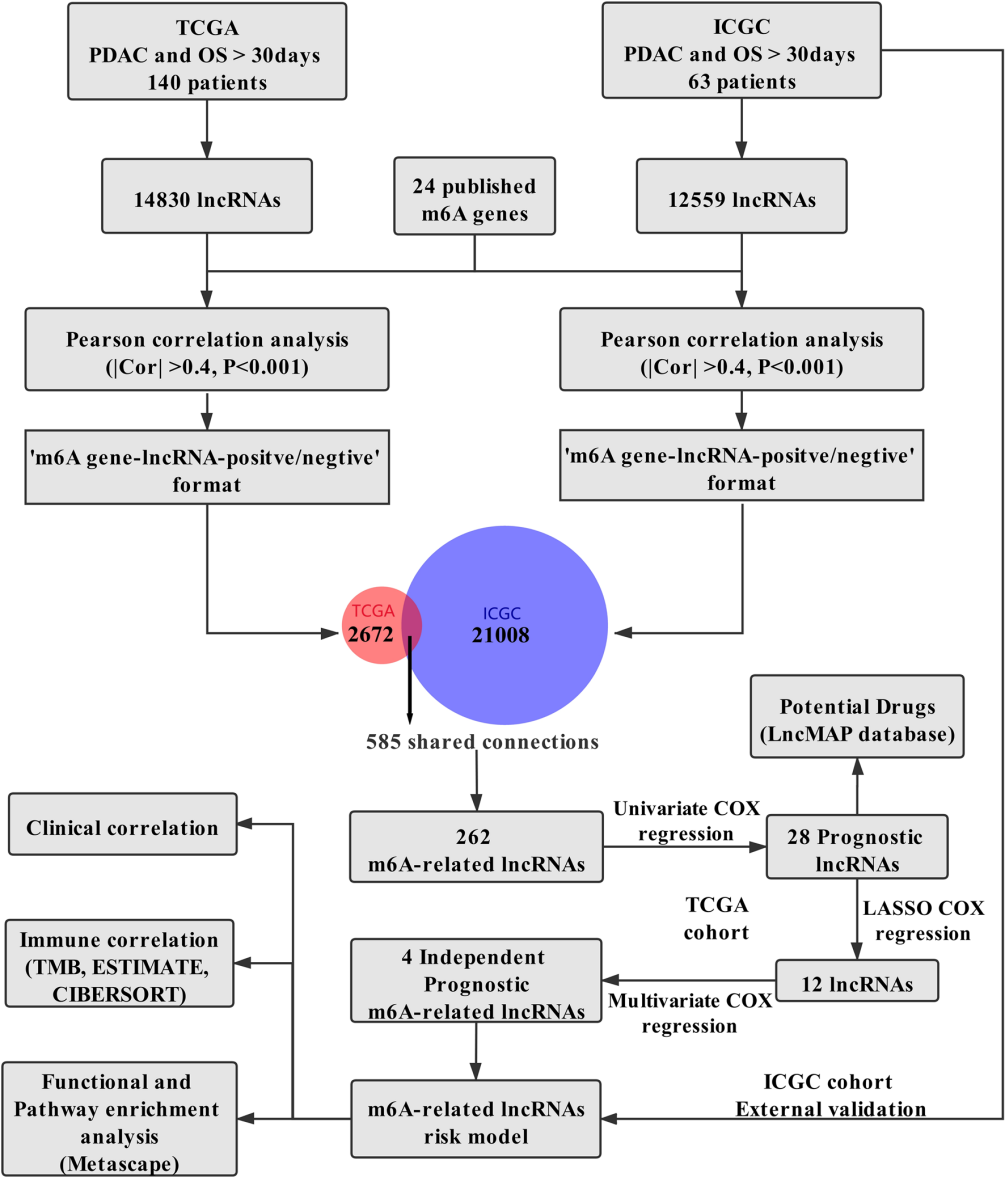
Flow chart of this study.

A flowchart of the study is shown in Fig. 1. For the TCGA training dataset, normalized RNA sequencing data [Fragments Per Kilobase of transcript per Million mapped reads (FPKM) normalized] and the corresponding clinicopathological data of PDAC were obtained from the Genomic Data Commons Data Portal (https://portal.gdc.cancer.gov/). To obtain an ICGC validation dataset, normalized RNA-seq data of PACA-AU and the corresponding clinicopathological data were downloaded from the ICGC Data Portal (https://dcc.icgc.org). Only the patients with histological confirmation in PDAC were included, patients with OS < 30 days or unknown OS status were excluded. Finally, we obtained the TCGA training cohort of 140 patients and the ICGC validation cohort of 63 patients. Moreover, on the basis of published literature, the expression data of 24 m6A-related genes were generated from the TCGA and ICGC datasets, including writers (*METTL3, METTL14, METTL16, RBM15, RBM15B, WTAP, VIRMA [KIAA1429], CBLL1,* and *ZC3H13*), erasers (*ALKBH5* and *FTO*) and readers (*YTHDF1, YTHDF2, YTHDF3, YTHDC1, YTHDC2, HNRNPC, HNRNPA2B1, IGF2BP1, IGF2BP2, IGF2BP3, FMR1, RBMX,* and *LRPPRC*). In our study, annotation of lncRNAs based on eight types of transcripts (lincRNA, antisense, 3prime overlapping ncRNA, processed transcript, sense overlapping, sense intronic, and macro lncRNA). Based on the Ensemble IDs and types of the transcript from the GENCODE website (https://www.gencodegenes.org/human/), 14,830 lncRNAs were identified in the TCGA cohort and 12,559 lncRNAs were identified in the ICGC cohort.

### Obtaining m6A-related lncRNAs

Pearson correlation analyses were applied to explore m6A-related lncRNAs (|Pearson R|> 0.4 and *p* < 0.001) in two cohorts. To ensure a more accurate corresponding relationship between m6A-related genes and lncRNAs, the results of correlation were built in the “m6A-related gene/lncRNA/positive or negative correlation” format (e.g., YTHDF1/ZFAS1/positive). The formatted results from the two cohorts were intersected to generate the 262 m6A-related lncRNAs from 582 correlations.

### Identifying prognostic m6A-related lncRNAs

We selected OS as the endpoint. In the TCGA training cohort, univariate Cox regression analysis was first conducted to identify the prognostic m6A-related lncRNAs, Next, the least absolute shrinkage and selection operator (LASSO) Cox regression analysis was performed using the R package “glmnet” through tenfold cross-validation. Finally, multivariate Cox regression analysis was conducted to identify the independent prognostic m6A-related lncRNAs. Based on the multivariate Cox regression result, an m6A-related lncRNA prognostic riskscore (m6A-LPR) model was developed for the PADC patients, each patient’s risk score was calculated by a combination of the expression levels of lncRNAs and multivariate Cox regression coefficients in the TCGA training cohort. We used the median risk score as the cut-off value to categorize the patients into high-risk groups and low-risk groups. The ICGC dataset was used as the validation cohort to verify the m6A-LPR. The time-dependent receiver operating characteristic (ROC) curves of the risk scores were conducted using the R package “timeROC” to evaluate the prognostic accuracy of m6A-LPR.

### Tumor immunity analyses

Stromal, immune, and estimate scores were calculated using the ESTIMATE algorithm^18^ which was generated from the expression data in the TCGA dataset (https://bioinformatics.mdanderson.org/public-software/estimate/), then we evaluated the differences in stromal, immune, and estimate scores between low- and high-risk groups of PDAC patients. Furthermore, to infer the relative abundance of tumor-infiltrating immune cells, CIBERSORT deconvolution algorithm^19^ was used, with the LM22 set representing 22 kinds of immune cells. We evaluated the differences in the immune infiltration of 22 immune cell subtypes between low- and high-risk groups of PDAC patients.

### Functional and pathway enrichment analysis

In the TCGA cohort, based on the m6A-LPR, differentially expressed genes (DEGs) between the high-risk groups and low-risk groups were identified using the R package “limma” (|log2(Fold change)|> 1 and False Discovery Rate (FDR) < 0.05). The 927 DEGs were obtained and imported into the “Metascape” website (https:matascape.org)^20^ for functional and pathway enrichment analysis, including Reactome Gene Sets, Canonical Pathways, Gene Ontology (GO) Biological Processes and Kyoto Encyclopedia of Genes and Genomes Pathway (KEGG pathway).

### Construct a ceRNA network for prognostic m6A-related lncRNAs

To elucidate the regulatory role of m6A-related lncRNAs, we constructed a ceRNA network by the prognostic m6A-related lncRNAs in m6A-LPR. DIANA-LncBase v3.0 (http://www.microrna.gr/LncBase)^21^ was utilized to predict miRNAs that interacted with lncRNAs, the miRNAs with high confidence level were selected. The relationship between miRNAs and target mRNAs was predicted in miRWalk3.0 database (http://mirwalk.umm.uni-heidelberg.de/)^22^, the mRNAs scored more than 0.95 and concurrently predicted by three databases (TargetScan, miRDB, and miRTarBase) were selected as target mRNAs, then the intersection of DEGs between the low- and high-risk groups with target mRNAs was put into the ceRNA network. Cytoscape software (version 3.8.2, http://www.cytoscape.org/)^23^ was used to visualize the lncRNA/miRNA/mRNA ceRNA network.

### Potential drugs for prognostic m6A-related lncRNAs

Drugs and affected lncRNAs were obtained from the LncMAP database (http://bio-bigdata.hrbmu.edu.cn/LncMAP/)^24^, which extracted drug-affected lncRNA expression profiles by reannotating the microarray data from the CMap database. We used the Drug-LncRNA Module of the LncMAP database to analyze the relationship between prognostic m6A-related lncRNAs and drugs. In the LncMAP database, the drug-lncRNA interaction was analyzed by Spearman’s correlation analysis between lncRNA expression levels and the IC50 values of the drug, and FDR less than 0.05 was considered significant. The m6A-related lncRNAs-drugs network was plotted by Cytoscape software (version 3.8.2, http://www.cytoscape.org/^23^.

### Statistical analysis

All statistical analyses and plots were performed using R Foundation Statistical software (version 4.0.2). The R packages “compareGroups_4.4.5”, “data.table_1.13.2”, “finalfit_1.0.2”, “ggpubr_0.4.0”, “ggsankey_0.0.9”, “glmnet_4.0-2”, “limma 3.46.0”, “maftools_2.6.0”, “reshape2_1.4.4”, “rtracklayer_1.49.5”, “survival_3.2-7”, “survminer_0.4.8”, “TCGAmutations_0.3.0”, “tidyverse_1.3.0”, and “vioplot_0.3.5” were used. Categorical variables were analyzed using the χ2 test or Fisher's exact test. Continuous variables were analyzed using Student's t-test or the Wilcoxon test. Survival was estimated using the Kaplan–Meier survival curves and compared using the log-rank test. Univariate, LASSO, and multivariate Cox regression analyses were performed to identify independent prognostic m6A-related lncRNAs and to develop the m6A-LPR. The hazard ratio (HR) and 95% confidence interval (CI) were calculated. Unless otherwise stipulated, two-tailed *p* < 0.05 was considered statistically significant.

## Results

### Identification of m6A-related lncRNAs in PDAC patients

The workflow was shown in Fig. 1. Cumulatively, 140 PDAC patients from the TCGA cohort and 63 PDAC patients from the ICGC cohort were included in our study; for whom, the baseline clinical features were presented in Table 1. Firstly, we identified 14,830 lncRNAs in the TCGA cohort and 12,559 lncRNAs in the ICGC cohort. Next, we extracted the expression data of lncRNAs and 24 m6A-related genes from the TCGA and the ICGC cohorts. Pearson correlation analyses were performed to identify m6A-related lncRNAs in two cohorts (|Pearson R|> 0.4 and p < 0.001). We obtained the 2672 correlations in the TCGA cohort and 21,008 correlations in ICGC cohorts, then the corresponding relationship between m6A-related genes and lncRNAs in two cohorts were intersected. Finally, 585 shared correlations (e.g., YTHDF1/ZFAS1/positive) were obtained in both two cohorts, because there were some duplicate lncRNAs in the 585 shared correlations, 262 unique m6A-related lncRNAs were extracted from the shared correlations after removing repetition.

**Table 1:** The baseline characteristics of patients included in this study.

Characteristics	TCGA (n, %)	ICGC (n, %)
**Age**		
> 65	69 (49.3%)	35 (55.6%)
≤ 65	71 (50.7%)	28 (44.4%)
**Gender**		
Female	66 (47.1%)	32 (50.8%)
Male	74 (52.9%)	31 (49.2%)
**Smoking**		
Yes	63 (45.0%)	–
No	50 (35.7%)	–
NA	27 (19.3%)	–
**Alcohol**		
Yes	79 (56.4%)	–
No	49 (35.0%)	–
NA	12 (8.57%)	–
**Diabetes**		
Yes	31 (22.1%)	–
No	85 (60.7%)	–
NA	24 (17.1%)	–
**Chronic pancreatitis**		
Yes	11 (7.86%)	–
No	100 (71.4%)	–
NA	29 (20.7%)	–
**Grade**		
G1	18 (12.9%)	1 (1.59%)
G2	81 (57.9%)	39 (61.9%)
G3-4	41 (29.3%)	22 (34.9%)
GX	–	1 (1.59%)
**Tumor location**		
Head of Pancreas	114 (81.4%)	–
Body & Tail of Pancreas	20 (14.3%)	–
Other	6 (4.29%)	–
**Tumorsize**		
≤ 2 cm	5 (3.57%)	–
> 2 cm & ≤ 4 cm	84 (60.0%)	–
> 4 cm	51 (36.4%)	–
**Stage**		
I	11 (7.86%)	–
II	123 (87.9%)	–
III/IV	6 (4.29%)	–
**T stage**		
T1	4 (2.86%)	–
T2	14 (10.0%)	4 (6.35%)
T3	119 (85.0%)	56 (88.9%)
T4	3 (2.14%)	1 (1.59%)
TX	–	2 (3.17%)
**N stage**		
N0	35 (25.0%)	16 (25.4%)
N1	105 (75.0%)	45 (71.4%)
Nx	–	2 (3.17%)
**M stage**		
M0	67 (47.9%)	2 (3.17%)
M1	3 (2.14%)	3 (4.76%)
Mx	70 (50.0%)	58 (92.1%)
**Survival status**		
Alive	57 (40.7%)	26 (41.3%)
Dead	83 (59.3%)	37 (58.7%)
**KRAS mutation**		
Yes	66 (47.1%)	–
No	53 (37.9%)	–
NA	21 (15.0%)	–
**TP53 mutation**		
Yes	69 (49.3%)	–
No	50 (35.7%)	–
NA	21 (15.0%)	–

### Identification of prognostic m6A-related lncRNAs

**Figure 2:**
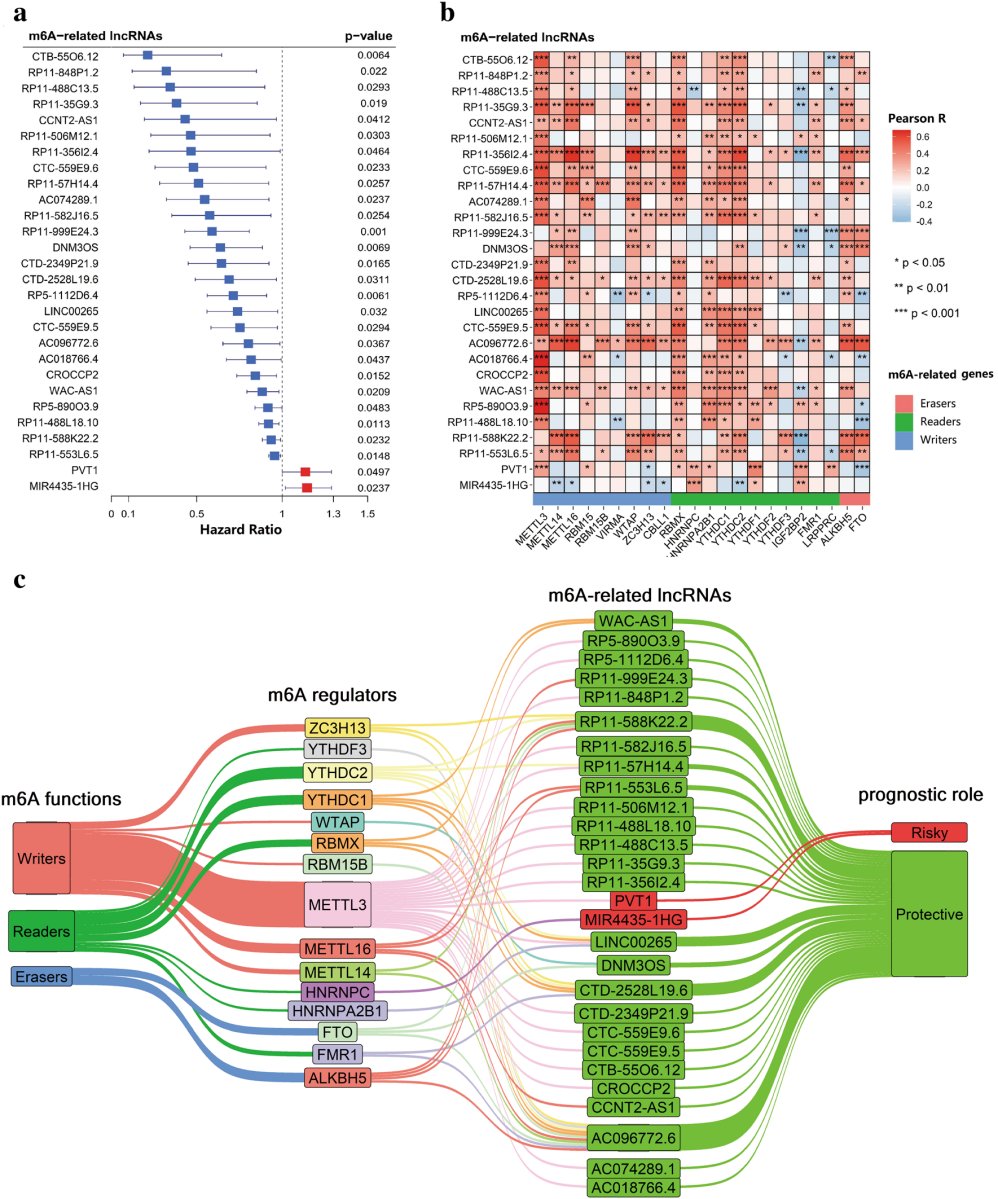
The 28 prognostic m6A-related lncRNAs. (**a**) The twenty-eight prognostic m6A-related lncRNAs in The Cancer Genome Atlas (TCGA) cohort. (**b**) Heatmap of the correlations between m6A-related genes and the 28 prognostic m6A-related lncRNAs. (**c**) Sankey diagram for m6A -related genes and m6A-related lncRNAs.

Univariate Cox regression was applied to identify prognostic m6A-related lncRNAs from the 262 m6A-related lncRNAs in the TCGA training cohort (p < 0.05). The result showed that 28 m6A-related lncRNAs were significantly associated with the OS (Fig. 2a), including 2 risky lncRNAs (PVT1 and MIR4435-1HG) and 26 protective lncRNAs. Among the 585 shared correlations (eg. YTHDF1/ZFAS1/positive) in both two cohorts, 22 m6A-related genes were extracted. Pearson correlation analyses were performed between the 28 lncRNAs and 22 m6A-related genes in the TCGA cohort, the results are shown in Fig. 2b. The 28 prognostic lncRNAs and their correlated m6A-related genes (m6A regulators) are shown in a Sankey diagram (Fig. 2c).

### Establishment of the m6A-LPR in the TCGA training cohort

**Figure 3:**
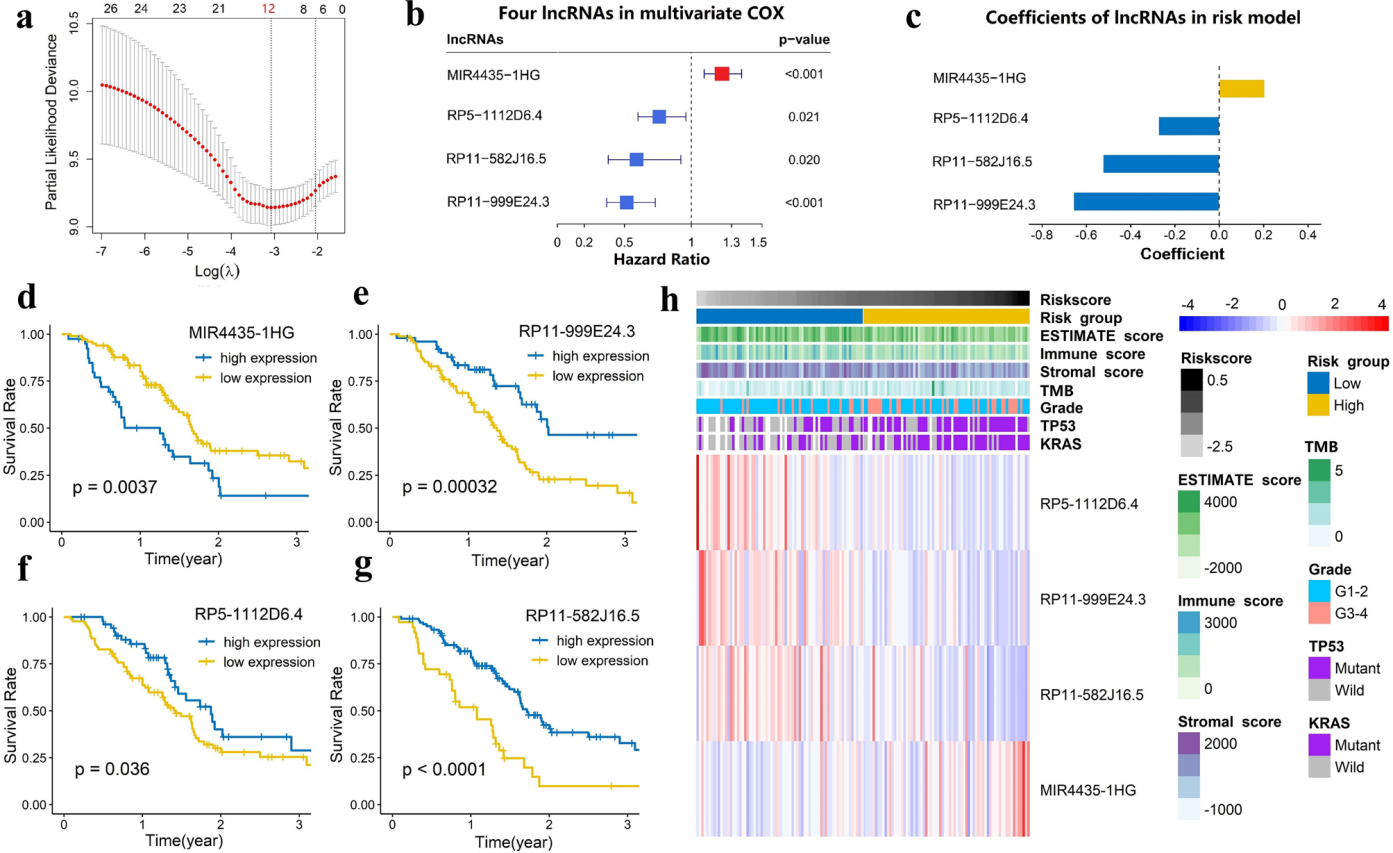
Four m6A-related lncRNAs in the risk model were identified by Least absolute shrinkage and selection operator (LASSO) regression and Multivariate cox regression. (**a**) LASSO regression was performed, calculating the minimum criteria. (**b,c**) Multivariate cox regression was performed, calculating the hazard ratio (HR), p-value **(b)**, and coefficients **(c)** for four m6A-related lncRNAs in the risk model. (**d–g**) Kaplan–Meier curves showing that patients with different expression levels of the four m6A-related lncRNAs had different overall survival. (**h**) Heatmap of the associations between the expression levels of the four m6A-related lncRNAs and clinicopathological features in The Cancer Genome Atlas (TCGA) cohort.

**Figure 4:**
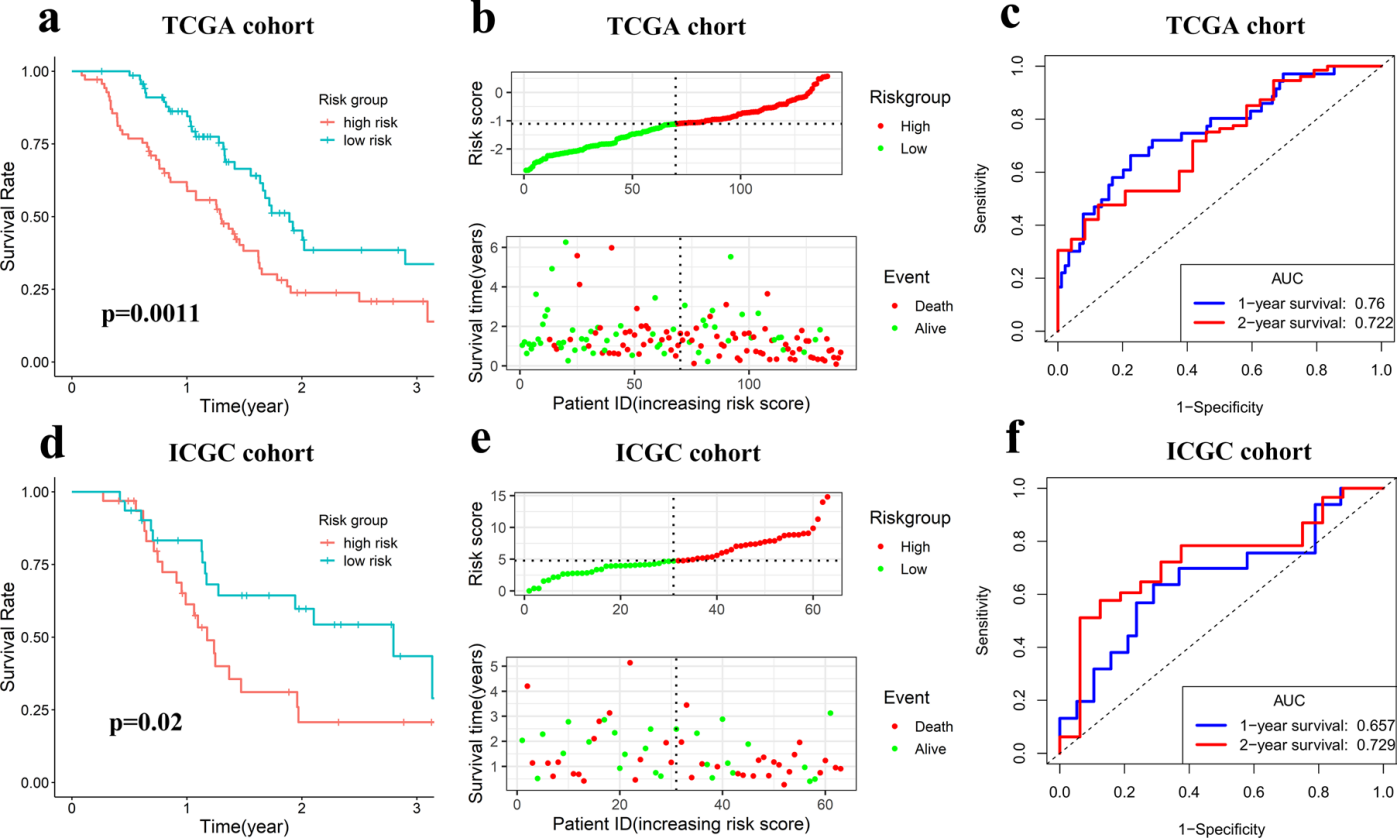
The m6A-related lncRNA prognostic riskscore (m6A-LPR) in the training cohort and validation cohort. (**a**) Kaplan–Meier curve showed that the high-risk group had worse overall survival than the low-risk group in the training cohort. (**b**) Distributions of risk scores based on the m6A-LPR and survival status of patients in the training cohort. (**c**) Receiver operating characteristic (ROC) curves of m6A-LPR for predicting the 1/2-year survival in the training cohort. (**d**) Kaplan–Meier curve showed that the high-risk group had worse overall survival than the low-risk group in the validation cohort. (**e**) Distributions of risk scores and survival status of patients in the validation cohort. (**f**) ROC curves of m6A-LPR for predicting 1/2-year survival in the validation cohort.

To construct the m6A-LPR for predicting the OS of PDAC patients, LASSO Cox analysis was performed on the 28 prognostic m6A-related lncRNAs in the TCGA cohort and 12 m6A-related lncRNAs were screened (Fig. 3a). Next, multivariate Cox proportional regression analysis was performed for analyzing these 12 m6A-related lncRNAs in the TCGA cohort to construct the m6A-LPR.The m6A-LPR comprising four m6A-related lncRNAs, including MIR4435-1HG, RP5-1112D6.4, RP11-582J16.5, and RP11-999E24.3, was developed through the summary of the expression values of these four m6A-related lncRNAs multiplied by corresponding coefficients derived from the above multivariable Cox regression analysis (Fig. 3b,c). The downregulated RP5-1112D6.4, RP11-582J16.5, and RP11-999E24.3 with HR < 1 were considered to be protective genes, while the upregulated MIR4435-1HG with HR > 1 was considered to be a risky gene. The Kaplan–Meier survival curves showed that higher expression of RP5-1112D6.4, RP11-582J16.5, and RP11-999E24.3 and lower expression of MIR4435-1HG were correlated with improved OS in the TCGA cohort (Fig. 3d–g). The expression of four m6A-related lncRNAs was also associated with the clinicopathological and immune signatures of PDAC, such as WHO grade, TP53 mutation status, KRAS mutation status, ESTIMATE score, immune score, stromal score, and tumor mutation burden (TMB) (Fig. 3h). The genomic information of these four m6A-related lncRNAs and the corresponding correlation with m6A regulators were presented in Table 2. The risk score for each patient was calculated by following formula based on m6A-LPR: [(0.205)*expression value of MIR4435-1HG]—[(0.525)*expression value of RP11-582J16.5]—[(0.658)*expression value of RP11-999E24.3]—[(0.275)*expression value of RP5-1112D6.4]. Based on the median value of risk scores, patients were categorized into low-risk and high-risk groups. Kaplan–Meier survival curves showed that PDAC patients with lower risk scores had better OS (p = 0.0011) (Fig. 4a). Survival status and risk score distributions were illustrated in Fig. 4b. The ROC curves demonstrated that m6A-LPR had a good performance for predicting OS in the TCGA cohort (1-year AUC = 0.760, 2-year AUC = 0.722; Fig. 4c).

**Table 2:** The genomic information of four prognostic m6A-related lncRNAs in m6A-related lncRNA prognostic riskscore (m6A-LPR) model.

	Ensembl ID	Aliases	Biotype	Location	Strand	m6A regulators (correlation)
MIR4435-1HG	ENSG00000172965	AGD2; MORRBID; LINC00978;MIR4435-2HG; lncRNA-AWPPH	Intronic	chr2:111,036,776–111,699,033	-	HNRNPC (positive)
RP11-582J16.5	ENSG00000253200	AC037459.3; Lnc-BIN3-1	Antisense	chr8:22,613,908–22,616,657	-	METTL3 (positive)
RP11-999E24.3	ENSG00000259969	Lnc-C14orf37-1; AL049838.1	Intergenic	chr14:57,993,545–57,994,525	-	ALKBH5 (positive)
RP5-1112D6.4	ENSG00000230177	Lnc-MFSD4B-1; AL080317.1	Intergenic	chr6:111,276,454–111,298,604	+	METTL3 (positive)

### Validation of the m6A-LPR in the ICGC Cohort

To further validate the robustness of m6A-LPR, the risk scores were calculated in the ICGC cohort using the above equation. Based on the median risk score, PDAC patients in the ICGC cohort were also divided into low- and high-risk subgroups. Consistent with the results in the TCGA training cohort: PDAC patients with lower risk scores had better OS in the ICGC validation cohort (p = 0.020) (Fig. 4d). Survival status and risk score distributions were shown in Fig. 4e. The ROC curves also demonstrated that m6A-LPR had a prognostic value for PDAC patients in the ICGC cohort (1-year AUC = 0.657, 2-year AUC = 0.729; Fig. 4f). These results showed that the m6A-LPR based prognostic signature had a robust and stable ability in prognosis prediction for PDAC.

### Principal component analysis

**Figure 5:**
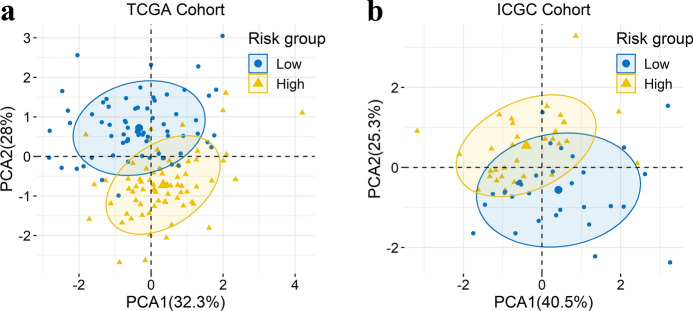
Principal component analysis (PCA) between the low- and high-risk groups based on the expression of the four m6A-related lncRNAs in the m6A-related lncRNA prognostic riskscore (m6A-LPR) in the training cohort (**a**) and the validation cohort (**b**).

Principal component analysis (PCA) was applied to evaluate the discrepancies between the low- and high-risk subgroups based on the expression of the four m6A-related lncRNAs in m6A-LPR (Fig. 5a,b). The results showed that the samples screened by the four m6A-related lncRNAs could clearly divide the whole patients into a low-risk and high-risk groups in both the TCGA and ICGC cohorts.

### Stratification analysis of the m6A-LPR in clinicopathological features

**Figure 6:**
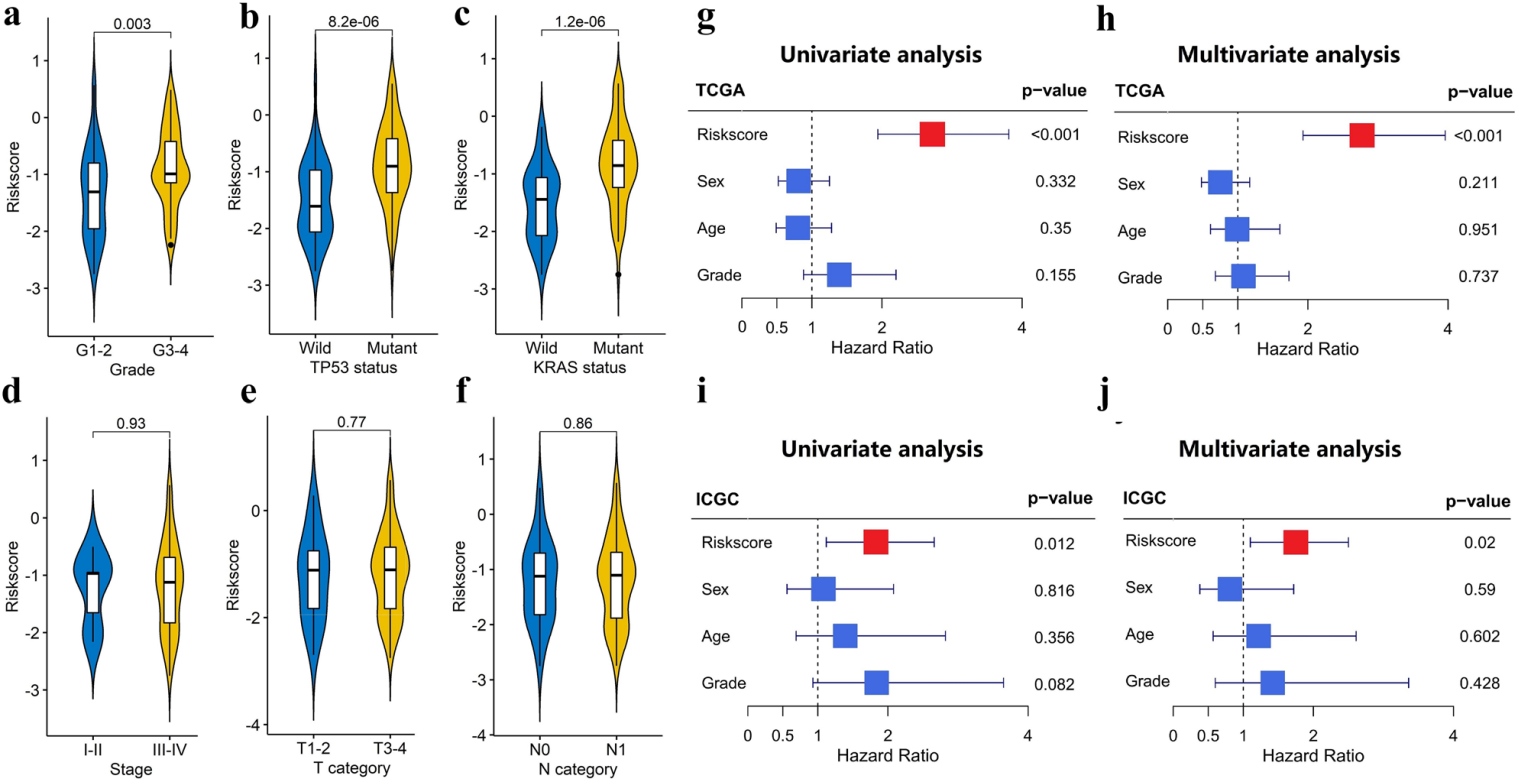
Stratification Analysis of the m6A-related lncRNA prognostic riskscore (m6A-LPR) in clinicopathological features. (**a–f**) Patients with different clinicopathological features (including grade, TP53 status, and KRAS status, but not stage, T category, and N category) had different levels of riskscore, calculated based on the m6A-LPR. (**g–j**) Univariate and multivariate analyses revealed that riskscore was an independent prognostic predictor in the training (**g,h**) and validation (**i,j**) cohorts.

PDAC patients with WHO grade III-IV, mutant TP53, and mutant KRAS (Fig. 6a–c) had higher risk scores, whereas the risk scores were not correlated with stage, T category, and N category (Fig. 6d–f). To evaluate whether m6A-LPR was an independent prognostic factor for PDAC patients, univariate and multivariate Cox analyses were performed. In the TCGA cohort, univariate Cox analysis showed that m6A-LPR was significantly associated with OS (HR: 2.72, 95% CI: 1.94–3.81, p < 0.001) and multivariate Cox analysis indicated that m6A-LPR was an independent predictor of OS (HR: 2.77, 95% CI: 1.93–3.96, p < 0.001; Fig. 6g,h). In the ICGC validation cohort, univariate and multivariate Cox analyses also indicated that m6A-LPR was an independent predictor of OS for PDAC patients (univariate: HR: 1.83, 95% CI: 1.12–2.66, p = 0.012; multivariate: HR: 1.75, 95% CI: 1.12–2.50, p = 0.020; Fig. 6i,j). These results demonstrated that m6A-LPR might be helpful for clinical **prognosis evaluation as an independent prognostic indicator.

### Stratification analysis of the m6A-LPR in immune features

**Figure 7:**
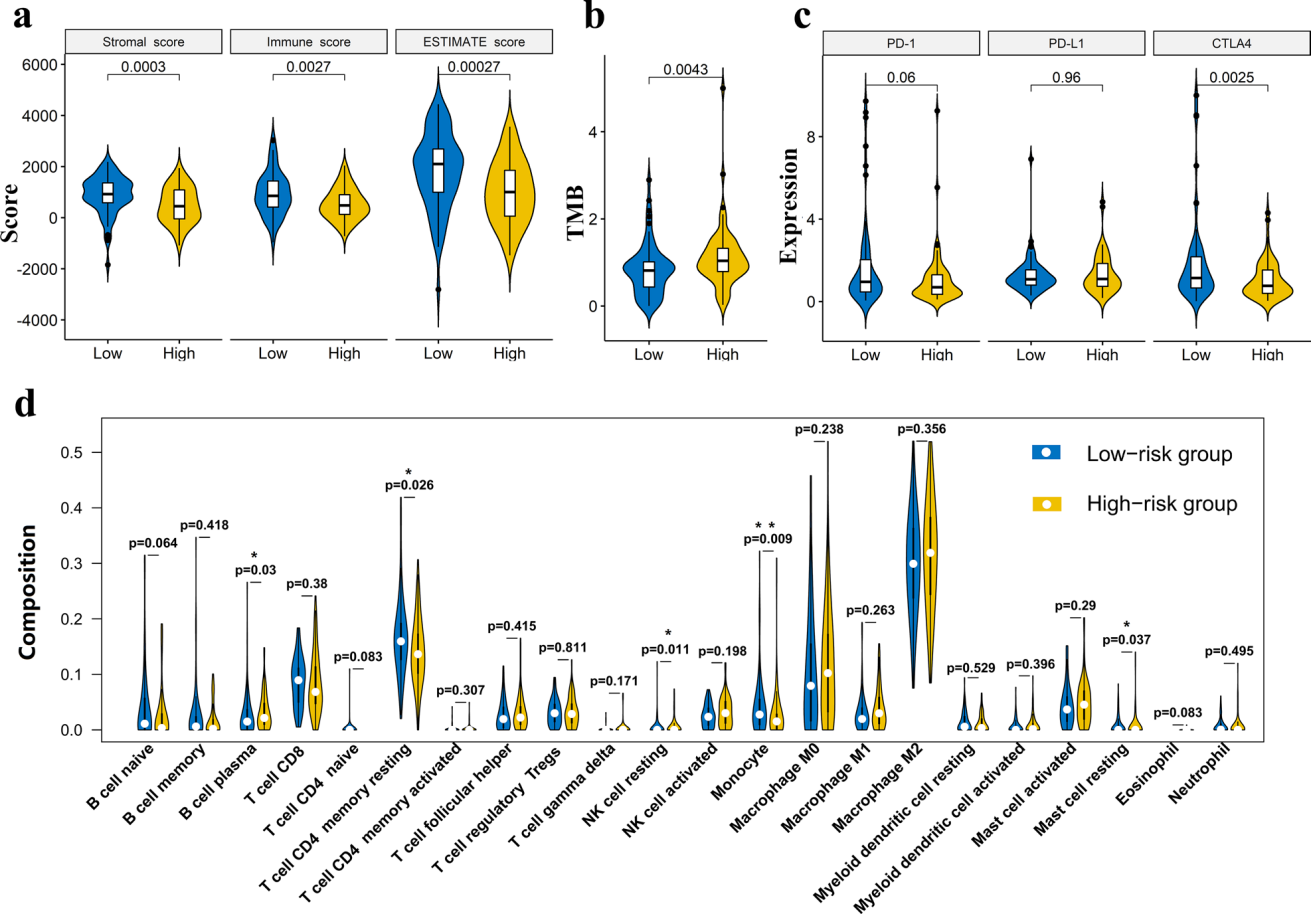
Stratification Analysis of the m6A-related lncRNA prognostic riskscore (m6A-LPR) in immune features. (**a,b**) The stromal score, immune score, tumor purity, and tumor mutation burden (TMB) significantly differ between the low- and high-risk groups based on the m6A-LPR in the training cohort. (**c**) Comparison of the expression pattern of immune checkpoint genes (PD1, PD-L1, and CTLA-4) between the low- and high-risk groups based on the m6A-LPR in the training cohort. (**d**) Relative infiltrating proportion of immune cells in low- and high-risk groups.

The relationship between m6A-LPR and tumor immunity was further evaluated. Tumor purity, TMB, Immune Checkpoint Molecules, and the infiltration level of the immune cells were estimated. PDAC patients in the high-risk group had remarkably lower stromal, immune, and ESTIMATE scores, indicating a lower level of stroma, immune cell infiltration, and tumor purity (Fig. 7a). Furthermore, PDAC patients in the high-risk group had significantly higher levels of TMB (Fig. 7b) and lower levels of CTLA4 expression (Fig. 7c). To further investigate the underlying molecular mechanisms of the m6A-LPR and its relevance to tumor immunity, the relative abundance of 22 tumor-infiltrating immune cells was assessed for each patient using CIBERSORT. The high-risk group was associated with significantly higher levels of plasma B cells and resting NK cells infiltration, and lower levels of infiltrating resting memory CD4 T cells, monocytes, and resting mast cells (Fig. 7d).

### Functional and pathway enrichment analysis

**Figure 8:**
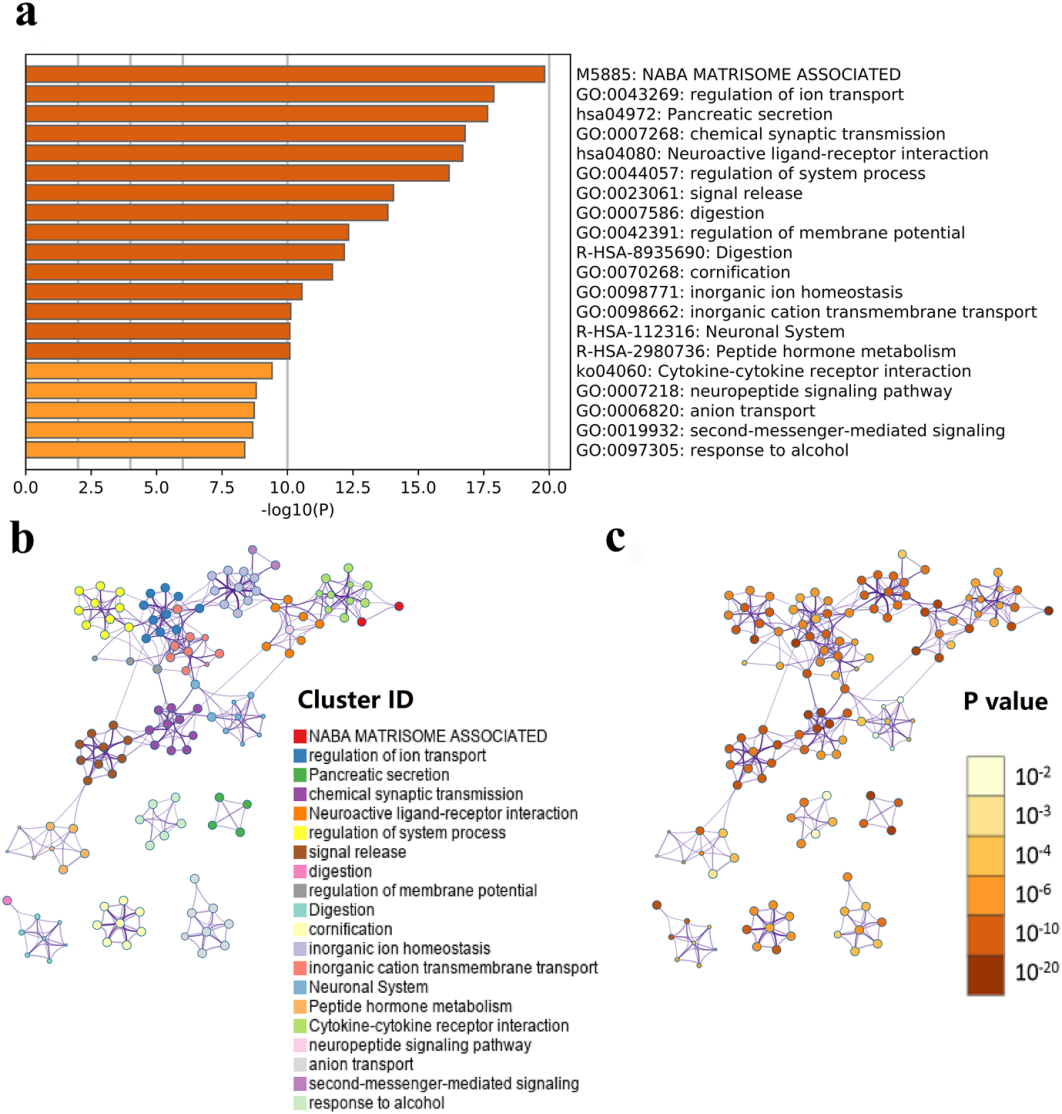
Functional analysis of 927 differentially expressed genes (DEGs) between the low- and high-risk groups. (**a**) Heatmap of enriched terms across the inputted gene list, colored according to p-value. Network of enriched terms colored according to cluster-ID (**b** nodes with the same cluster-ID are typically close to each other) and p-value (**c** terms with more genes tend to have higher p-values).

To explore the potential biological processes and pathways of the molecular discrepancy between the low-risk and high-risk groups, 927 differential expression genes (DEGs) were identified between the low-risk and high-risk groups in the TCGA cohort (|log2 (fold change) |> 1 and p < 0.05) (Additional file 1 Table S1). Functional and pathway enrichment analysis indicated these DEGs were mainly enriched in these aspects: digestion, neuronal system and peptide hormone metabolism (Reactome Gene Sets); NABA matrisome-associated (Canonical Pathways); pancreatic secretion, neuroactive ligand-receptor interaction, and cytokine-cytokine receptor interaction (KEGG Pathways); regulation of ion transport, chemical synaptic transmission, regulation of system process, signal release, neuropeptide signaling pathway, and second-messenger-mediate signaling (GO Biological Processes) (Fig. 8a–c). These results could give us some insights into the potential molecular mechanisms of the m6A-LPR.

### Construction of the ceRNA network

**Figure 9:**
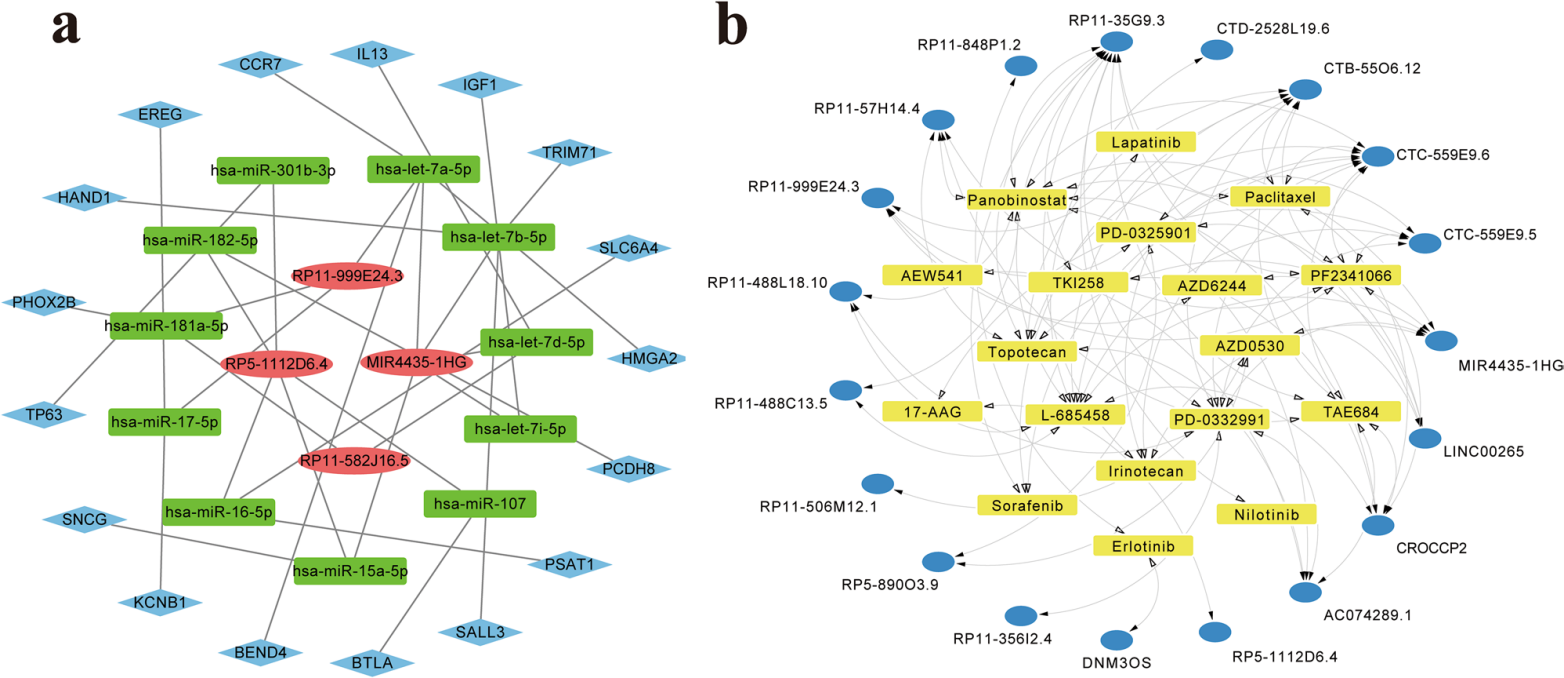
The ceRNA network for four m6A-related lncRNAs in the m6A-related lncRNA prognostic riskscore (m6A-LPR) and the drug-lncRNA network for 28 prognostic m6A-related lncRNAs. (**a**) The ceRNA network of the four m6A-related lncRNAs (red) and their target miRNAs (green) and mRNAs (blue). (**b**) Potential targeted drugs (yellow) for 28 prognostic m6A-related lncRNAs (blue) in the Drug-LncRNA Module of the LncMAP database (False Discovery Rate (FDR) < 0.05).

To further elucidate how the m6A-related lncRNAs in m6A-LPR regulate mRNA by sponging miRNAs in PDAC, we constructed a ceRNA network based on the m6A-related lncRNAs and DEGs. Four lncRNAs in m6A-LPR were extracted from the DIANA-LncBase v3.0 and 90 pairs of interaction between the 4 lncRNAs and 72 miRNAs were identified. Then we used miRWalk3.0 database which included three databases (miRTarBase, miRDB, and TargetScan) to predict target mRNAs based on the 72 miRNAs, and a total of 1103 mRNAs were identified. Then 927 DEGs were intersected with 1103 target mRNAs and 17 intersected target mRNAs were selected into the ceRNA network. Ultimately, based on the seventeen intersected target mRNAs, four lncRNAs, eleven miRNAs, and seventeen mRNAs were included in our ceRNA network (Fig. 9a).

### Explore potential drugs that have a therapeutic effect on PDAC

From the Drug-LncRNA Module of the LncMAP database, we obtained 304,304 drug-lncRNA interaction pairs. A total of 28 prognostic m6A-related lncRNAs were then imported into the database to predict the potential drugs of the genes, and 75 drug-lncRNA interactions were extracted when FDR < 0.05. The network including 18 prognostic m6A-related lncRNAs and 18 drugs was identified (Fig. 9b). The five most interactions with prognostic m6A-related lncRNAs drugs were Panobinostat, L-685458, Palbociclib, Crizotinib, and TAE684.

## Discussion

PDAC is an extremely challenging disease since only 80% of patients with PDAC are not amenable to surgery at diagnosis^3^. Recently, with the benefit of high-throughput sequencing, studies to explore the molecular markers of PDAC at the molecular and cellular level have got breakthroughs, which will be helpful for increasing the prognostic accuracy and introducing potential therapeutic targets for PDAC. An increasing number of studies indicated that epigenetic alterations can largely affect cancer progression^25^. Among that, m6A modification is the most common epigenetic methylated modification of mRNAs and ncRNAs^4^, it has been confirmed to play critical regulatory roles in the modification of tumor RNAs^5^. Aberrant lncRNAs had been found to be an important role in the carcinogenicity of PDAC^9,15–17^. However, few studies have been conducted to investigate the mechanisms underlying how lncRNAs are regulated by m6A modification to involve in the onset and development of PDAC. Therefore, we tried to identify m6A-related lncRNAs through bioinformatics analysis from two public datasets. Twenty-eight m6A-related lncRNAs had prognostic value, and four of them were screened to build an m6A-LPR for predicting the OS of PDAC patients. Furthermore, we explored the correlation of m6A-LPR with clinicopathological and immune features of PDAC and tried to find potential target drugs for prognostic m6A-related lncRNAs.

Previous studies had shown that the stability of lncRNAs is enhanced by the accumulation of m6A modifications^26^, with the binding of low-complexity proteins^27^, interactions with m6A readers^9^, and additional regulatory mechanisms. Recent studies had demonstrated that m6A modification can regulate oncogenesis and tumor progression in PDAC^8–13^, but it is still unclear how m6A modification affects the occurrence and progression of PDAC in a lncRNA-dependent pattern. The m6A eraser ALKBH5 could demethylate the lncRNA KCNK15-AS1 and inhibit KCNK15-AS1-mediated pancreatic cancer cell motility^28^. The m6A reader IGF2BP2 could interact with the lncRNA DANCR and promote cancer stemness-like properties and pancreatic cancer pathogenesis^9^. Studies had shown that m6A modification of lncRNAs may have an effect on the occurrence and progression of cancer and lncRNAs may act as a target for m6A regulators to influence aggressive tumor progression. Based on these evidence, we should pay more attention to the interactions between lncRNAs and m6A modifications to identify potential therapeutic targets or prognosis markers of cancers.

We identified 28 prognostic m6A-related lncRNAs from the TCGA dataset, and four of them were included in the m6A-LPR and validated in the ICGC dataset. The ROC curves demonstrated that m6A-LPR had a good performance for predicting OS in the TCGA cohort (1-year AUC = 0.760, 2-year AUC = 0.722) and ICGC cohort (1-year AUC = 0.657, 2-year AUC = 0.729). To evaluate the clinical utility of the m6A-LPR, we combined it with clinicopathological characteristics and performed univariate and multivariate Cox regression analyses. We found that the m6A-LPR was an independent prognostic factor in the TCGA and ICGC cohorts, implying that the m6A-LPR could be used to predict OS in patients with PDAC independently and reliably. Furthermore, we performed a stratification analysis of the m6A-LPR in clinicopathological features, and patients in the high-risk subgroup had a higher WHO grade and proportion of TP53 and KRAS mutation. It also demonstrated the clinical utility of the m6A-LPR.

In the m6A-LPR model, RP5-1112D6.4, RP11-582J16.5, and RP11-999E24.3 were protective genes, MIR4435-1HG was a risky gene. MIR4435-1HG is highly expressed and acts as a risky gene in renal cell carcinoma, and it promotes cell proliferation, migration, and invasive capacity of renal carcinoma cells^29^. It has been reported that MIR4435-1HG (also termed LINC00978) promotes the progression of hepatocellular carcinoma by inhibiting p21 and E-cadherin expression via EZH2-mediated epigenetic silencing^30^ and promotes cell proliferation and tumorigenesis via regulating microRNA-497/NTRK3 axis in gastric cancer^31^, there is no report regarding PDAC. The other three lncRNAs (RP5-1112D6.4, RP11-582J16.5, and RP11-999E24.3) have not been reported in the literature and their functions are unknown. As a result, we hope that our findings may aid in the identification of prognostic lncRNAs that m6A regulators may target, offering insight into their possible involvement in PDAC tumorigenesis and progression.

In many types of tumors, such as skin melanoma, breast cancer, colon cancer, and non-small cell lung cancer, tumor immune infiltrating cells account for a high proportion based on lncRNA sequencing data^32^. The lncRNA lnc-EGFR can stimulate T-regulatory cells differentiation thus promoting hepatocellular carcinoma immune evasion^33^. LINC00473 can drive the progression of pancreatic cancer via upregulating PD-L1^34^. Even though lncRNA therapeutics have only become the focus of investigations in the past decade and no lncRNA-targeting therapeutics have entered clinical development so far^35^, a recent study^36^ summarized the most investigated lncRNAs which are involved in cancer immunoediting, TME modulation, and immunotherapy resistance, it indicated that lncRNAs have the potential value as promising immunotherapeutic targets. Therefore, we explored the relationship between m6A-LPR and tumor immunity using four m6A-related lncRNAs in m6A-LRP, and found that m6A-LPR for PDAC was associated with TMB, tumor purity, and the infiltration of immune cell subtypes. In our study, the level of resting memory CD4 T cells, monocytes, and resting mast cells were low whereas the level of plasma B cells and resting NK cells were high in the high-risk group, the result implies that imbalance of immune cell may reduce the survival rate of patients in the high-risk group. Our findings may contribute to new understanding of the molecular biological mechanism of lncRNAs, as well as provide novel model for prognosis prediction and therapy decision-making for patients with PDAC.

We performed functional analysis of the DEGs in low- and high-risk patients stratified by m6A-LPR to explore the role of four m6A-related lncRNAs in PDAC. The analysis revealed that four m6A-related lncRNAs were significantly enriched in cell ion exchange (regulation of ion transport, inorganic ion homeostasis, and anion transport) and biological processes of signaling pathways (neuroactive ligand-receptor interaction, signal release, cytokine-cytokine receptor interaction, neuropeptide signaling pathway, and second-messenger-mediated signaling). The result implied that the m6A-LPR might be related to maintaining cellular homeostasis and cell injury, thus affecting the progression of the tumor. The findings are probably used to develop new targeted anti-cancer therapies for PDAC if the hypothesis can be proved.

This study included two PDAC datasets, the TCGA and ICGC datasets, and m6A-LPR containing four prognostic m6A-related lncRNAs were built in the TCGA dataset and validated in the ICGC dataset, but there were several limitations in our study. First, the interactions between lncRNAs and m6A-related genes were obtained from two datasets in our study, they should be validated by more independent cohorts and confirmed through in vivo and in vitro experiments. Second, in two datasets, the clinical data are incomplete and exist selection bias. Third, because PDAC has a very poor survival rate and the sample sizes in the two cohorts are small, we chose 2-year OS as the endpoint. The prognostic ability of m6A-LPR for OS > 2 years needs further validation. Forth, traditional statistical analyzes were used to construct and validate the prognostic risk model of m6A-LPR. Although these methods had been utilized and validated in many studies, it is crucial to improve further studies with more advanced methodologies. To further verify our bioinformatics results, in-depth studies on the m6A-LPR containing four prognostic m6A-related lncRNAs, including molecular mechanisms and functional experiments, are needed.

## Conclusion

In summary, we built and validated a prognostic model of m6A-LPR containing four prognostic m6A-related lncRNAs. The m6A-LPR not only provides additional information for PDAC prognostic analyses but also affects the immunity of PDAC. Further studies are needed to validate our model and to explore the molecular mechanism and function of m6A-LPR in the regulation of anti-tumor immunity, our results may provide some clues for further studies.

## Supplementary Information

Supplementary Information.

## Data Availability

TCGA training data was downloaded from the TCGA database (http://cancergenome.nih.gov) under the accession number TCGA-PAAD. ICGC validation data was downloaded from the ICGC database (https://dcc.icgc.org) under the accession number PACA-AU.

## Abbreviations

PDAC: Pancreatic ductal adenocarcinoma
m6A: N6-methylandenosine
TCGA: The Cancer Genome Atlas
ICGC: International Cancer Genome Consortium
LASSO: Least absolute shrinkage and selection operator
m6A-LPR: M6A-related lncRNAs prognostic riskscore
OS: Overall survival
ROC: Receiver operating characteristic
mRNAs: Messenger RNAs
lncRNAs: Long non-coding RNAs
FPKM: Fragments Per Kilobase of transcript per Million
DEGs: Differentially expressed genes
FDR: False Discovery Rate
GO: Gene Ontology
KEGG: Kyoto Encyclopedia of Genes and Genomes
HR: Hazard ratio
CI: Confidence interval
TMB: Tumor mutation burden
ceRNA: Competing endogenous RNAs
